# Epidemiological, Virulence, and Antibiotic Resistance Analysis of *Klebsiella pneumoniae*, a Major Source of Threat to Livestock and Poultry in Some Regions of Xinjiang, China

**DOI:** 10.3390/ani14101433

**Published:** 2024-05-10

**Authors:** Gongmingzhu Hou, Sajjad Ahmad, Yanfang Li, Duo Yan, Shuhan Yang, Siqi Chen, Zhengqing Qiu, Xingyu Yu, Nana Li, Yang Li, Yan Liang, Qingwen Leng, Yonggang Qu

**Affiliations:** College of Animal Science and Technology, Shihezi University, Shihezi 832003, China; hougongmingzhu@foxmail.com (G.H.); ahmadsajjad@stu.shzu.edu.cn (S.A.); yanfangli@shzu.edu.cn (Y.L.); yanduo315@163.com (D.Y.); yangshuhan0311@163.com (S.Y.); barbatos39@foxmail.com (S.C.); q772913867@163.com (Z.Q.); yuxingyu117@foxmail.com (X.Y.); linana1217@foxmail.com (N.L.); liyang9902@foxmail.com (Y.L.); 18199661608@163.com (Y.L.)

**Keywords:** *Klebsiella pneumoniae*, livestock, poultry, multilocus sequence typing, *wzi* gene sequencing, virulence gene, antibiotic resistance

## Abstract

**Simple Summary:**

The current study aimed to investigate the virulence and resistance genes associated with *Klebsiella pneumoniae* isolated from the farms of four animal species in selected areas of Xinjiang, China, encompassing cows, sheep, chickens, and pigs. The sensitivity to antibiotics was evaluated. Genetic diversity was assessed using *wzi* allele typing and multilocus sequence typing. The findings shed light on the antibiotic resistance patterns and genetic diversity among *K. pneumoniae* isolates originating from different animal sources, highlighting the potential for inter-species transmission of this bacterium.

**Abstract:**

*Klebsiella pneumoniae* (*K. pneumoniae*) is recognized as a zoonotic pathogen with an increasing threat to livestock and poultry. However, research on *K. pneumoniae* of animal origin remains limited. To address the gap, a comprehensive investigation was carried out by collecting a total of 311 samples from the farms of four animal species (dairy cow, chicken, sheep, and pig) in selected areas of Xinjiang, China. Isolates were identified by *khe* gene amplification and 16S rRNA gene sequencing. Genotyping of *K. pneumonia* isolates was performed using *wzi* typing and multilocus sequence typing (MLST). PCR was employed to identify virulence and resistance genes. An antibiotic susceptibility test was conducted using the Kirby–Bauer method. The findings revealed an isolation of 62 *K. pneumoniae* strains, with an average isolation rate of 19.94%, with the highest proportion originating from cattle sources (33.33%). Over 85.00% of these isolates harbored six virulence genes (*wabG, uge, fimH, markD, entB,* and *ureA*); while more than 75.00% of isolates possessed four resistance genes (*bla*_TEM_*, bla_SHV_, oqxA*, and *gyrA*). All isolates exhibited complete resistance to ampicillin and demonstrated substantial resistance to sulfisoxazole, amoxicillin/clavulanic acid, and enrofloxacin, with an antibiotic resistance rate of more than 50%. Furthermore, 48.39% (30/62) of isolates were classified as multidrug-resistant (MDR) strains, with a significantly higher isolation rate observed in the swine farms (66.67%) compared to other farms. Genetic characterization revealed the classification of the 62 isolates into 30 distinct *wzi* allele types or 35 different sequence types (STs). Notably, we identified *K. pneumoniae* strains of dairy and swine origin belonging to the same ST42 and wzi33-KL64 types, as well as strains of dairy and chicken origin belonging to the same wzi31-KL31-K31 type. These findings emphasize the widespread occurrence of drug-resistant *K. pneumoniae* across diverse animal sources in Xinjiang, underscoring the high prevalence of multidrug resistance. Additionally, our results suggest the potential for animal-to-animal transmission of *K. pneumoniae* and there was a correlation between virulence genes and antibiotic resistance genes. Moreover, the current study provides valuable data on the prevalence, antibiotic resistance, and genetic diversity of *K. pneumoniae* originating from diverse animal sources in Xinjiang, China.

## 1. Introduction

*K. pneumoniae* is a Gram-negative bacterium commonly found in various environmental niches, including water, soil, plants, and the mucosal surfaces of both humans and animals [1]. In recent years, there has been a notable increase in the morbidity and mortality associated with *K. pneumoniae* infections in livestock, poultry, and wildlife worldwide, leading to significant detrimental effects [2]. In animals, *K. pneumoniae* infections manifest in diverse forms, including as mastitis and respiratory infections in dairy cows, pneumonia and upper respiratory infections in sheep, and pneumonia, mastitis, and septicemia in swine [3,4,5]. Additionally, poultry can suffer from pneumonia, liver abscesses, enteritis, and septicemia caused by *K. pneumoniae* [6]. Based on virulence and pathogenicity, *K. pneumoniae* can be divided into classical *K*. *pneumoniae* (cKP) and hypervirulent *K*. *pneumoniae* (hvKP) [7]. Several virulence factors contribute to the pathogenicity of *K. pneumoniae*, including fimbriae, capsular polysaccharides (CPS), lipopolysaccharides (LPS), and iron carriers [7]. LPS shields bacteria from host immune responses, while fimbriae facilitate bacterial adhesion and biofilm formation, thereby enhancing the virulence [8]. CPS, forming a protective polysaccharide layer around the bacterial cell, is crucial for *K. pneumoniae* virulence. In both cKP and hvKP strains of *K*. *pneumoniae*, genes responsible for capsule synthesis are situated on chromosomal operons known as CPS [9]. The regulation of CPS synthesis is primarily governed by key genes such as *rmpA* and *magA*. Notably, a considerable proportion of hvKP strains harbor either *rmpA* or *rmpA2* genes [10]. Moreover, the acquisition of iron is crucial for bacterial growth and replication. Within *K*. *pneumoniae*, four distinct iron-absorbing molecules, or iron carriers, have been identified: enterobactin, yersinobactin, salmochelin, and aerobactin. While enterobactin is prevalent in both cKP and hvKP strains and serves as the principal iron uptake system, the other three iron carriers are more frequently encountered in hvKP strains [11,12].

However, due to the misuse of antibiotics, MDR *K. pneumoniae* is proliferating at an alarming rate, presenting a significant challenge to antibiotic treatment [13,14]. Both animals and their products can serve as reservoirs for MDR strains, and *K*. *pneumoniae* poses a potential risk of inter-species transmission between humans and animals, as well as among animals themselves [15]. For instance, Dereeper et al. [16] identified multiple high-risk clonal strains of *K*. *pneumoniae* of human origin in livestock and pets in Guadeloupe. However, research on animal-to-animal transmission is limited. In China, it was found that *K*. *pneumoniae* isolated from milk samples from dairy farms in Jiangsu and Shandong provinces showed high levels of resistance to tetracyclines, chloramphenicol, and aminoglycosides [17] and carried multiple antibiotic resistance genes. *K. pneumoniae* isolated from pig farms in Jilin Province, China, were mainly ST11, ST2230, and ST2234, many of which were the same as the strains ST found in hospital-acquired infections and they showed high resistance to quinolones, β-lactams, and aminoglycosides [18]. Even, tigecycline-resistant *K*. *pneumoniae* was isolated from cecum samples collected from a chicken farm in Jiangsu Province, China [19]. It can be seen that *K*. *pneumoniae* infections are more serious in different animal farms in the Chinese region. However, studies on *K*. *pneumoniae* of animal origin in Xinjiang are limited; therefore, the present study aimed to investigate the prevalence, antibiotic resistance, and carriage of virulence and resistance genes of *K*. *pneumoniae* in major livestock and poultry, including dairy cows, sheep, swine, and chickens in the Xinjiang region of China, to provide a theoretical basis for the prevention and treatment of *K. pneumoniae* infections in Xinjiang. In addition, genotyping was performed using multilocus sequence typing (MLST) and *wzi* typing to assess genetic diversity and explore transmission among different animals. This study will contribute to the epidemiological data concerning *K. pneumoniae* of animal origin in China.

## 2. Materials and Methods

### 2.1. Sample Collection

Large-scale farms, spanning dairy cows, sheep, chickens, and swine, were selected for sample acquisition in different regions of Xinjiang. Samples were collected as specified below. Cows with clinical mastitis (CM) can be diagnosed by at least 1 of the following symptoms: elevated uterine temperature, blood clots in the milk, and uterine swelling [20]. Subclinical mastitis (SCM) was judged according to the Chinese Agricultural Standard NY/T 2692-2015 (National Standard of the People’s Republic of China, 2015) [21], and was detected by the somatic cell count (SCC) method; if there were no pathologic changes visible to the naked eye, but the SCC in the milk was >500,000 cells/mL, the cow had subclinical mastitis. Milk samples were collected by the dairy worker after alcohol sterilization and the first three milk streams were discarded and milk samples were collected using fifty-milliliter sterile centrifuge tubes. Sheep or swine with symptoms such as shallow shortness of breath, dyspnea, purulent and mucopurulent nasal discharge, loss of appetite, and emaciation were categorized as sick animals with respiratory disease. Nasal and anal swabs were collected by inserting the swab into the animal’s nasal or anal cavity and rotating it 2 to 3 times. Environmental samples were collected randomly and feed samples were collected from feeding troughs. All samples were labelled accordingly, placed in sampling containers at 4 °C, and transported to the laboratory for bacterial isolation and identification. Detailed sampling information is shown in Table 1. 

Two large-scale dairy farms (Farm A and Farm B) were selected, about 180 km apart. Farm A is located in Urumqi, Xinjiang, China, with 1000 Holstein cows, while Farm B is located in the Tacheng region of Xinjiang, China, with 800 Holstein cows. Both farms share the same management style, with cows fed a total mixed ration (TMR), cows milked three times a day, and sawdust used for bedding. The sheep farm is located in Shihezi City, Xinjiang, China, with 500 heads of Kazakh sheep, one of the local sheep breeds in Xinjiang. Captive breeding was used and barley and soybean meal were fed. The chicken farm is located in Changji, Xinjiang, China. The farm currently has 10 chicken sheds with about 6000 Hyland Brown Shell egg-laying hens in each building and adopts a three-dimensional farming model with automatic lamp-controlled lighting and an automatic water supply system. The swine farm, also located in Changji, Xinjiang, China, but about 170 km away from the chicken farm, has 1400 New American Duroc pigs. Swine farms are intensively managed.

### 2.2. Isolation and Identification of K. pneumoniae

All samples were inoculated into 5 mL of brain heart infusion (BHI) broth (Haibo Biotech, Qingdao, China) and incubated for 18–24 h at 37 °C in incubator shakers (Crystal, Addison, TX, USA). Subsequently, broth cultures were streaked on MacConkey Inositol Adonitol Carbenicillin (MIAC) agar (Haibo Biotech, Qingdao, China) using an inoculating loop and incubated at 37 °C for 12 to 18 h. Single colonies exhibiting suspicious characteristics—pink coloration, mucus-like consistency, and rounded morphology—on MIAC agar were selected and purified by streaking three consecutive times on MIAC agar.

Gram staining was performed on the purified bacteria using a Gram stain kit (Solarbio Technology, Beijing, China), and biochemical identification was carried out using a biochemical identification tube (Hangzhou Microbial Reagent Co., Hangzhou, China). Subsequently, the genomic DNA of the purified bacteria was extracted using a DNA extraction kit (Tiangen, Beijing, China), and further identification was conducted using PCR targeting *khe* (GenBank accession no. CP035202.1) and 16S rRNA genes [22,23]. Positive clones were sequenced by Youkang Biologicals (Urumqi, China). *K*. *pneumoniae* CMCC 46117 (*khe* gene positive) was used as a positive control strain.

### 2.3. Capsular Polysaccharide Characterization and Multilocus Sequence Typing

The detection of capsular polysaccharide phenotypes of *K. pneumoniae* using *wzi* gene sequencing has been documented [24]. Molecular typing of *K. pneumoniae* was conducted using the MLST method, which involves PCR amplification of seven highly conserved housekeeping genes (*gapA, infB, mdh, phoE, pgi, rpoB, tonB*). The PCR protocol involved an initial denaturation step at 95 °C for 3 min, followed by 30 cycles of denaturation at 94 °C for 25 s, annealing for 25 s at the specified temperature, and extension at 72 °C for 1 min, with a final extension step at 72 °C for 5 min. Subsequently, all positive PCR products were sequenced using the Sanger method. The resulting sequences were then submitted to the website (https://bigsdb.pasteur.fr/klebsiella/, accessed on 8 June 2023) to obtain the *wzi* allele type, the allele numbers of the seven housekeeping genes for MLST, and the corresponding sequence type (ST). Sanger sequencing was performed by General Biologicals (Anhui, China). (Refer to Table S1 for details).

### 2.4. Virulence Gene Detection

The detection of carriage of 12 virulence genes in all *K. pneumoniae* isolates was conducted using PCR, following established protocols outlined in relevant literature [25,26,27]. These virulence genes included lipopolysaccharide-related genes (*uge, wabG*), capsular polysaccharide synthesis and synthesis regulation-related genes (*rmpA, magA*), fimbriae synthesis-related genes (*fimH, mrkD*), iron uptake system genes (*entB, kfu, iroN, icuA*), an urease-related gene (*ureA*), and a allantoin-related gene (*allS*). Primer sequences and related information are provided in Table S2.

The PCR protocol involved an initial denaturation step at 95 °C for 3 min, followed by 30 cycles of denaturation at 94 °C for 25 s, annealing for 25 s at the specified temperature, and extension at 72 °C for 1 min, with a final extension step at 72 °C for 10 min. The PCR products were then analyzed on a 1% agarose gel to visualize the amplified DNA fragments.

### 2.5. Antibiotic Resistance Gene Detection

Following established methodologies described in pertinent literature [27,28,29], PCR was utilized to identify the presence of 21 antibiotic genes belonging to six distinct classes in all *K. pneumoniae* isolates. These genes included β-lactams (*bla*_IMP_, *bla*_VIM_, *bla*_OXA-48_, *bla*_NDM_, *bla*_KPC_, *bla*_DHA_, *bla*_FOX_, *bla*_CTX-M-2_, *bla*_SHV_, and *bla*_TEM_), Quinolones (*oqxA, aac(6′)-Ib-cr, qnrA,* and *gyrA*), Amphenicols (*floR*), Sulfonamides (*sul1* and *sul2*), Tetracyclines (*tetA* and *tetB*), and Aminoglycosides (*aadA1* and *aacC2*). Primer sequences and relevant information are provided in Table S2. The PCR reaction conditions were consistent with those outlined in Section 2.4.

### 2.6. Antibiotic Susceptibility Test

Antibiotic susceptibility testing was performed on Mueller–Hinton agar (MHA) using the Kirby–Bauer disk diffusion method [30]. A total of 15 antibiotics were tested, including ampicillin (AMP, 10 μg), florfenicol (FFC, 30 μg), enrofloxacin (ENR, 5 μg), ciprofloxacin (CIP, 5 μg), gentamicin (CN, 10 μg), kanamycin (KAN, 30 μg), meropenem (MEM, 10 μg), imipenem (IPM, 10 μg), tetracycline (TCY, 30 μg), sulfisoxazole (SF, 300 μg), cotrimoxazole (SXT, 1.25/23.75 μg), ceftazidime (CAZ, 30 μg), cefotaxime (CTX, 30 μg), ceftriaxone (CRO, 30 μg), and amoxicillin/clavulanic acid (AMC, 20/10 μg). The antimicrobial disks were obtained from Microbial Reagent (Hangzhou Microbial Reagent Co., Hangzhou, China). The quality control strains were *K. pneumoniae* ATCC 700603 and *Escherichia coli* ATCC 25922. Interpretation of the diameters of the zone of inhibition was based on the recommendations of the Institute of Clinical and Laboratory Standards [31]. Isolates were categorized as MDR if they were resistant to ≥3 different antibiotic classes [32].

### 2.7. Statistical Analysis

All statistical analyses were performed using SPSS 22.0 software (IBM, Armonk, NY, USA) and GraphPad Prism version 9.0.1 software. The chi-square test (χ2) was used to compare the statistical significance between groups. *p* < 0.05 indicated significant difference and *p* < 0.01 indicated highly significant difference. The Spearman correlation coefficient (r), a non-parametric indicator of the degree of correlation between two variables, has a value between −1 and 1. Correlation was assessed by chi-square test and calculation of Spearman’s rank correlation coefficient (r). |r| ≥ 0.8 indicated high correlation, 0.5 ≤ |r| < 0.8 indicated moderate correlation, 0.3 ≤ |r| < 0.5 indicated low correlation, and |r| < 0.3 indicated no correlation.

## 3. Results

### 3.1. Prevalence of K. pneumoniae in Different Animal Farms

**Figure 1:**
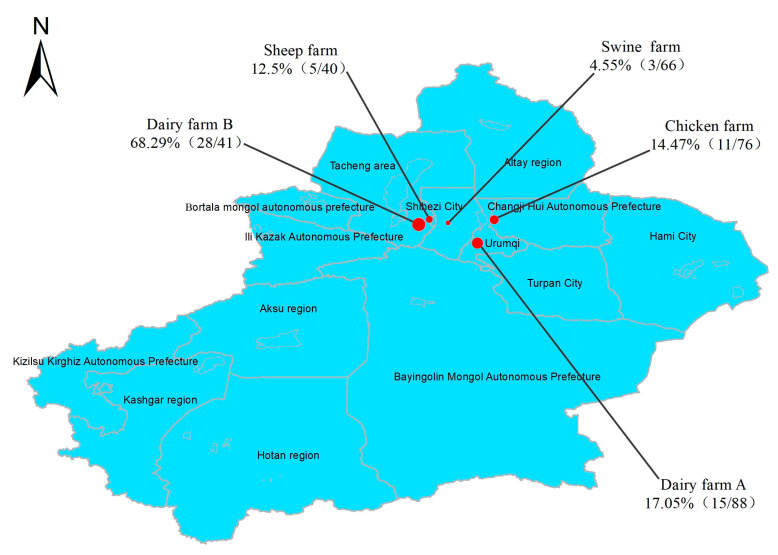
Percentage of *K*. *pneumoniae* in different farm samples. The size of the circle indicates the high or low detection rate of *K*. *pneumoniae*.

**Figure 2:**
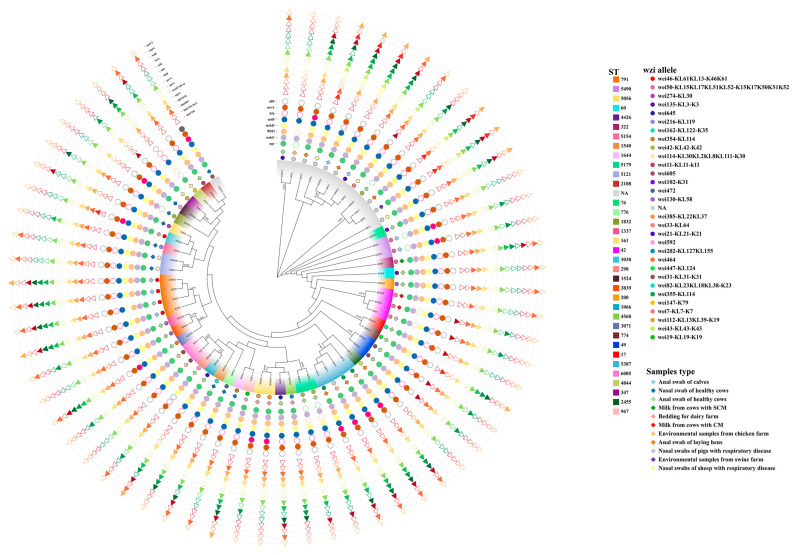
Sequence type (ST) phylogenetic tree of *K. pneumoniae*, *wzi* allele typing, virulence gene detection, resistance gene detection. Colored solid shapes indicate that the gene is detected and hollow shapes indicate that the gene is not detected. NA: unknown typing.

After biochemical characterization, the isolates exhibited a negative non-dynamic, indocyanine substrate test and a positive urease test, citrate test, VP test, glucose gas production test, and lactose test, and these results were consistent with the biochemical characteristics of *K*. *pneumoniae.* With an average isolation rate of 19.94%, a total of 62 strains of *K. pneumoniae* were recovered and identified from 311 samples through *khe* gene amplification and 16S rRNA gene sequencing (see Figures S1 and S2). The distribution of *K. pneumoniae* isolates varied across different animal sources. The highest percentage of *K. pneumoniae* isolates originated from dairy sources (43 out of 129 samples, accounting for 33.33%), especially Dairy Farm B where the isolation rate was as high as 68.29%. Conversely, the lowest percentage of *K. pneumoniae* isolates was derived from swine sources (4.55%). Intermediate percentages of *K. pneumoniae* isolates were obtained from chicken sources (14.47%) and sheep sources (12.5%) (see Figure 1). Notably, higher isolation rates of *K. pneumoniae* were observed in anal and nasal swab samples obtained from healthy cattle. Additionally, *K. pneumoniae* was also isolated from various environmental samples, including five strains from environmental samples from the chicken farm, four strains from dairy farm bedding, and one strain from environmental samples from the swine farm (see Figure 2).

### 3.2. Capsular Serotyping and Multilocus Sequence Typing

Based on the *wzi* gene sequences, the 62 isolates were classified into 30 distinct *wzi* allele types (see Figure 2 and Table S3). The predominant model, wzi46-KL61KL13-K46K61, accounted for 8.06% (5/62) of the isolates. Additionally, 19 *wzi* allele types were identified, each representing 1.61% (1/62) of the isolates. Furthermore, the 62 *K. pneumoniae* strains were assigned to 35 different sequence types (STs) based on the analysis of seven conserved alleles of the MLST gene (see Figure 2 and Table S4). The most prevalent STs were ST791 and ST5387, each comprising 6.45% (4/62) of the isolates, followed by ST42 (4.84%, 3/62). Additionally, ST49, ST5179, ST5490, ST5121, ST161, and ST6085 accounted for 3.23% (2/62) of the isolates. The remaining 26 STs were represented by a single strain.

A sequence type (ST) phylogenetic tree of 63 *K. pneumoniae* strains was constructed, and the results are shown in Figure 2. The isolates from the Xinjiang region exhibited a variety of capsular types and sequence types. It was observed that strains with the same serotype could be part of different genotypes, and conversely, strains with the same genotype could display different serotypes. For instance, both ST290 and ST1524 share the capsular type wzi592. Notably, *K. pneumoniae* isolates with capsular type wzi31-KL31-K31 were found in dairy and chicken farm samples. In contrast, capsular type wzi33-KL64 and ST42 strains were identified in dairy and pig farm samples. These findings suggest that *K. pneumoniae* has the potential to be transmitted between animals of different species.

**Figure 3:**
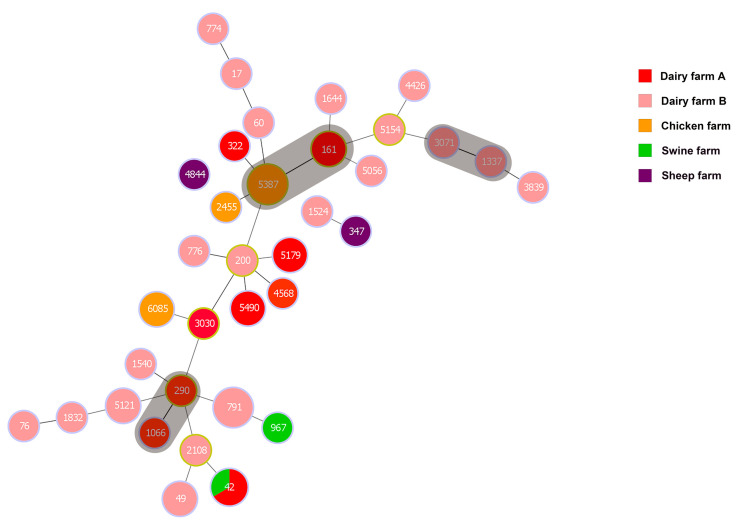
Minimum spanning tree (MST) of *K. pneumoniae*. The gray area represents a clonal complex and the number represents ST. Each ST is grouped by different sample types.

A minimum spanning tree was constructed for 49 strains of *K. pneumoniae* except for 13 untyped strains (see Figure 3). The tree revealed three clonal complexes (CC) out of 35 ST types: ST161 and ST5387, ST3071 and ST1337, ST290 and ST1066. STs in the latter two CCs were exclusively from the same dairy farm. Whereas, ST161 from the first clonal complex was obtained from *K. pneumoniae* in dairy farm bedding in Urumqi, ST5387 was obtained from anal swab samples of healthy laying hens and environmental fecal samples from the chicken farms. In addition, ST42 was detected in samples from both the swine and dairy farms. This all indicates the homology of *K. pneumoniae* from different animal sources. Meanwhile, isolates from the sheep farms could be found to be genetically distant from isolates from other farms, and *K. pneumoniae* of dairy origin had rich genomic diversity.

### 3.3. Virulence Gene Detection

Eight out of the 12 virulence genes tested positive via PCR and they were *wabG, uge, fimH, mrkD, entB, ureA, kfu,* and *allS*. The detection rates of the lipopolysaccharide-related genes (*wabG, uge*), fimbriae synthesis-related genes (*fimH, mrkD*), iron uptake system gene (*entB*), and urease-related gene (*ureA*) were all above 85%. The iron uptake system gene (*kfu*) exhibited a detection rate of 20.97%, whereas the allantoin-related gene (*allS*) showed the lowest detection rate at 1.61%. Notably, high-virulence-associated genes such as *rmpA, magA, iroN*, and *iucA* were not detected.

Among the *K. pneumoniae* isolates, the number of virulence genes detected ranged from two to seven. The most commonly detected combination involved six virulence genes (*uge-wabG-fimH-mrkD-entB-ureA*), found in 39 isolates (62.90%, 39/62). The distribution of virulence genes among *K. pneumoniae* isolates from different animal sources exhibited considerable consistency. However, the iron uptake system gene *kfu* was exclusively detected in isolates from dairy and chicken origins, albeit with significant differences in carriage rates. Additionally, the allantoin-related gene *allS* was solely detected in isolates from dairy sources. Further details can be found in Table 2.

### 3.4. Antibiotic Susceptibility Testing

**Figure 4:**
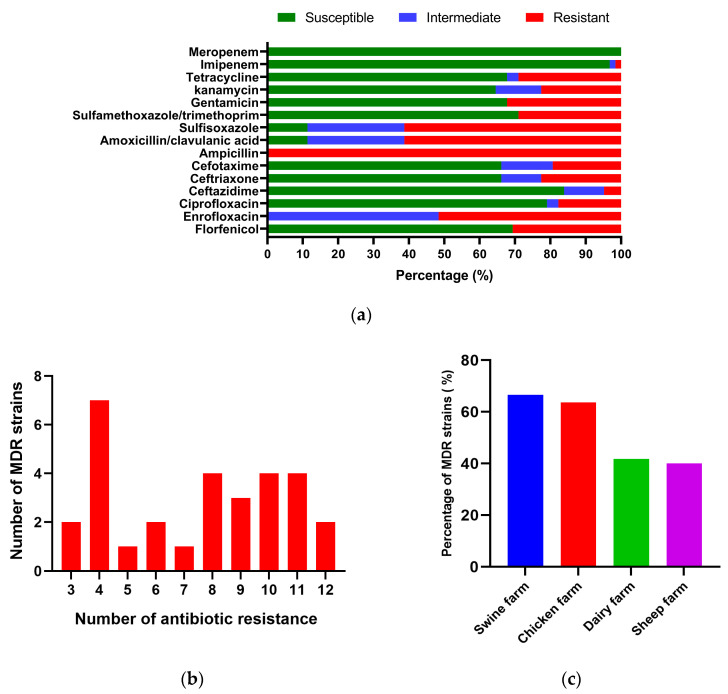
Antibiotic resistance of *K*. *pneumoniae* isolates: (**a**) Resistance rate of *K. pneumoniae* to 15 antibiotics. (**b**) Statistics on the number of MDR *K. pneumoniae* resistant to antibiotics. (**c**) Percentages of MDR *K. pneumoniae* isolates resistant in different animal farms.

The susceptibility of 62 *K. pneumoniae* isolates to 15 common antibiotics is illustrated in Figure 4a. The isolates of *K. pneumoniae* were highly susceptible to meropenem (100.00%) and imipenem (96.77%), followed by ceftazidime (83.87%) and ciprofloxacin (79.03%). However, *K. pneumoniae* was completely resistant to ampicillin, 61.29% to both sulfisoxazole and amoxicillin/clavulanic acid, 51.61% to enrofloxacin, and resistance to florfenicol, gentamicin, tetracycline, cefotaxime, cotrimoxazole, kanamycin, and ceftriaxone ranged from 17.74 to 32.26%. As shown in Table 3, *K. pneumoniae* isolated from the chicken farms showed higher resistance to all antibiotics than other animal farms except three antibiotics, namely amoxicillin/clavulanic acid, kanamycin, and sulfisoxazole; whereas, *K. pneumoniae* of sheep origin showed the lowest resistance rate. Among these isolates, varying degrees of resistance were observed, with 30 isolates categorized as MDR, accounting for 48.39% of the total, and two isolates were resistant to 12 antibiotics (see Figure 4b). In addition, MDR was prevalent in all four animal sources of *K. pneumoniae*, with a higher incidence of MDR *K. pneumoniae* in swine and chicken sources (see Figure 4c).

### 3.5. Antibiotic Resistance Genes

The 62 isolates of *K. pneumoniae* were assessed for the presence of 21 resistance genes, revealing a total of 15 resistance genes across six antibiotic classes (see Table 4). Notably, the detection rates of quinolone resistance genes *gyrA* and *oqxA* exceeded 85.00%, while the detection rates of β-lactam resistance genes *bla*_TEM_ and *bla*_SHV_ surpassed 75.00%. Conversely, the detection rates of the remaining 11 resistance genes ranged from 4.84% to 50.00%. 

Significant variations were observed in the carriage of antibiotic resistance genes among *K. pneumoniae* isolates originating from different animal sources. Carriage of antibiotic resistance genes was significantly higher in isolates of swine origin compared to isolates from other animal sources.

### 3.6. Correlations between Antibiotic Resistant Phenotypes and Resistant Genes

**Figure 5:**
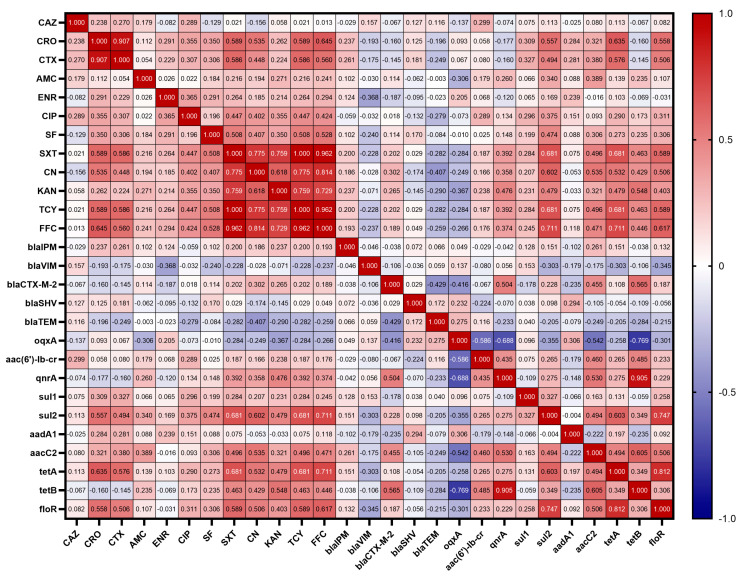
Correlation of antibiotic resistance phenotypes with antibiotic resistance genes. The intensity of the color indicates the numerical value of the correlation coefficient (r).

The resistance genes carried by 62 strains of *K*. *pneumoniae* were correlated with the resistance phenotypes of the antibiotics, and the results are shown in Figure 5. Among β-lactams, antibiotic resistance phenotypes and resistance genes showed no significant correlation. However, interestingly, CRO showed a low positive correlation with the sulfonamide resistance gene *sul1* (r = 0.309), a moderate positive correlation with *sul2* (r = 0.557), a low positive correlation with the aminoglycoside resistance gene *aacC2* (r = 0.321), a moderate positive correlation with the tetracycline resistance gene *tetA* (r = 0.635) and the amphenicol resistance gene *floR* (r = 0.558); CTX showed a low positive correlation with both *sul1* and *sul2* (r = 0.327, r = 0.494), a low positive correlation with *aacC2* (r = 0.380), a moderate positive correlation with *tetA* (r = 0.576), and a moderate positive correlation with *floR* (r = 0.506); and AMC showed a low negative correlation with the quinolone resistance gene *oqxA* (r = −0.306), a low positive correlation with *sul2* (r = 0.340), and a low positive correlation with *aacC2* (r = 0.389). 

Among quinolones, antibiotic resistance phenotypes and resistance genes also showed no significant correlation. However, CIP was found to show a low positive correlation with the amphenicol resistance gene *floR* (r = 0.311) and a low positive correlation with the sulfonamide resistance gene *sul2* (r = 0.375); ENR showed a low negative correlation with the β-lactam gene *bla*_VIM_ (r = −0.368).

Among sulfonamides, SF showed a low positive correlation with *sul2* (r = 0.474) and SXT showed a moderate positive correlation with *sul2* (r = 0.681). SF also showed a low positive correlation with the amphenicol resistance gene *floR* (r = 0.306) and a low positive correlation with the aminoglycoside resistance gene *aacC2* (r = 0.306); SXT showed a moderate positive correlation with *floR* (r = 0.589), a moderate positive correlation with the tetracycline resistance gene *tetA* (r = 0.681), a low positive correlation with *tetB* (r = 0.463), a low positive correlation with *aacC2* (r = 0.496), and a low positive correlation with the quinolone resistance gene *qnrA* (r = 0.392). 

Among aminoglycosides, CN showed a moderate positive correlation with *aacC2* (r = 0.535) and KAN showed a low positive correlation with *aacC2* (r = 0.321). In addition, CN was moderately positively correlated with the amphenicol resistance gene *floR* (r = 0.506), moderately positively correlated with the tetracycline resistance gene *tetA* (r = 0.532), and lowly positively correlated with *tetB* (r = 0.429), moderately positively correlated with the sulfonamide resistance gene *sul2* (r = 0.602), and lowly positively correlated with the quinolone resistance gene *qnrA* (r = 0.358), lowly negatively correlated with the β-lactam gene *bla*_TEM_ (r = −0.407), and lowly positively correlated with *bla*_CTX_ (r = 0.302); KAN showed a low positive correlation with *floR* (r = 0.403), a low positive correlation with tetA (r = 0.479), a moderate positive correlation with *tetB* (r = 0.548), a low positive correlation with *sul2* (r = 0.479), a low positive correlation with *qnrA* (r = 0.476), and a low negative correlation with *oqxA* (r = −0.367).

Among tetracyclines, TCY showed a moderate positive correlation with *tetA* (r = 0.681) and a low positive correlation with *tetB* (r = 0.463). Meanwhile, TCY was moderately positively correlated with the amphenicol resistance gene *floR* (r = 0.589), lowly positively correlated with the aminoglycoside resistance gene *aacC2* (r = 0.496), moderately positively correlated with the sulfonamide resistance gene *sul2* (r = 0.681), and lowly positively correlated with the quinolone resistance gene *qnrA* (r = 0.392).

Finally, among the amphenicols, FFC showed a moderate positive correlation with *floR* (r = 0.617). In addition, FFC showed a moderate positive correlation with the tetracycline resistance gene *tetA* (r = 0.711), a low positive correlation with *tetB* (r = 0.446), a low positive correlation with the aminoglycoside resistance gene *aacC2* (r = 0.471), a moderate positive correlation with the sulfonamide resistance gene *sul2* (r = 0.711), and a low positive correlation with the quinolone resistance gene *qnrA* (r = 0.374).

### 3.7. Correlation Analysis of Virulence and Antibiotic Resistance

**Figure 6:**
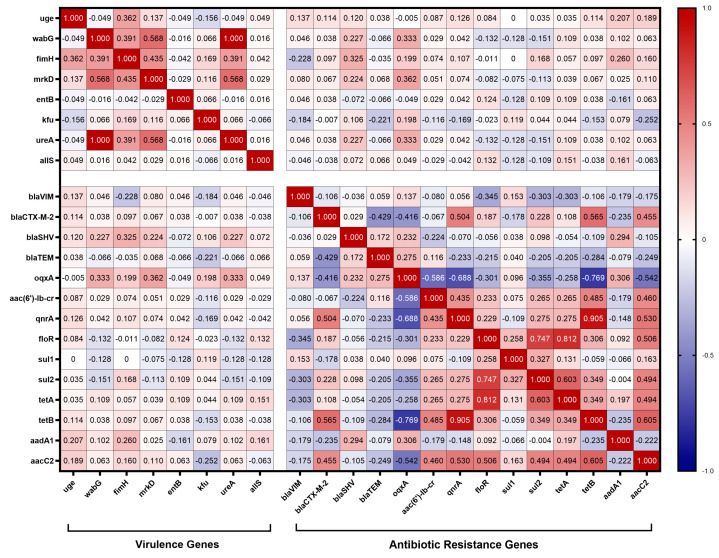
Correlation between virulence genes and antibiotic resistance genes. The intensity of the color indicates the numerical value of the correlation coefficient (r).

To reveal the interaction between the presence of virulence genes and antibiotic resistance genes, in this study, correlation coefficients were calculated between the virulence genes and antibiotic resistance genes carried by 62 strains of *K. pneumoniae*. The results, shown in Figure 6, revealed a weak correlation between the virulence genes carried by *K. pneumoniae* isolates and antibiotic resistance genes. Specifically, there was a low positive correlation between the fimbriae synthesis-related gene *fimH* and the β-lactam resistance gene *bla*_SHV_ (r =0.325); and there was a low positive correlation between the quinolone resistance gene *oqxA* and the lipopolysaccharide-related gene *wabG*, the fimbriae synthesis-related gene *mrkD*, and the urease-related gene *ureA* (r = 0.333, 0.362, and 0.333). Other than that, no significant association was found between other virulence genes and antibiotic resistance genes.

**Figure 7:**
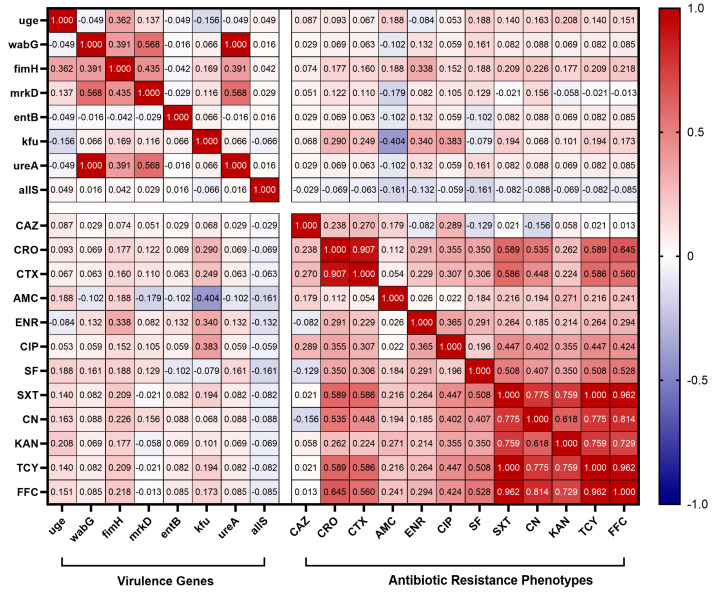
Correlation between virulence genes and antibiotic resistance phenotypes. The intensity of the color indicates the numerical value of the correlation coefficient (r).

The correlation between virulence genes carried by 62 *K. pneumoniae* isolates and antibiotic resistance phenotype was also analyzed in this study. The results are shown in Figure 7, where it can be observed that the carriage rate of the iron uptake system gene *kfu* of *K. pneumoniae* isolates showed a low negative correlation with the resistance rate of AMC (r = −0.404) and a low positive correlation with the resistance rates of ENR (r = 0.340) and CIP (r = 0.383). In addition, the *fimH* carrier rate was significantly low and positively correlated with ENR resistance (r = 0.338).

## 4. Discussion

Xinjiang, known for its abundant natural resources, is a significant pastoral region in China where agriculture plays a key role in the economy. Livestock farming in the region mainly focuses on beef cattle, meat sheep, and dairy farming. However, dairy cow mastitis is a common issue worldwide, affecting dairy farming by reducing milk production and quality. Among the various pathogens that cause mastitis in dairy cows, *K. pneumoniae* is identified as a primary environmental factor [33]. Similarly, respiratory ailments persist as a looming threat to the well-being of small ruminants (such as sheep and goats), exacting significant tolls in terms of diminished productivity, decreased offspring rates, and heightened mortality [34]. Notably, a study examining bacterial pathogens in sheep afflicted with respiratory disease in northwestern Egypt identified *K. pneumoniae* as the predominant causative agent [35]. Furthermore, in Norwegian poultry, healthy turkeys proved to be a greater reservoir of *K. pneumoniae* than healthy broilers [36]. Additionally, *K. pneumoniae* has been implicated in diminishing egg production in laying hens, consequently inflicting economic losses. Despite its pronounced impact on animal health and agricultural productivity, research on *K. pneumoniae* has predominantly concentrated on human infections, with comparatively scant attention directed toward infections and multidrug resistance in *K. pneumoniae* of animal origin. This paucity of data impedes clinical management and underscores the necessity for further exploration into this aspect of *K. pneumoniae* research. 

This study investigated the prevalence, capsular typing, sequence typing, virulence genes, resistance genes, and antibiotic susceptibility of *K. pneumoniae* in four species of animal farms (dairy cows, sheep, chickens, and swine). The detection rates of *K. pneumoniae* varied significantly between farms, with the highest total detection rate observed in dairy farms (dairy farm A and B) at 33.33%, notably higher than the other types of animal farms. Moreover, dairy farm B (68.29%) exhibited a significantly higher detection rate compared to dairy farm A (17.05%). This phenomenon is similar to the results of Cheng et al. who showed differences in the detection rate of *K. pneumoniae* in two dairy farms in southern and northern China [37]. In the current study, 10 strains of *K. pneumoniae* were also isolated from dairy farm bedding, and environmental samples from chicken and pig farms, which confirms previous reports [38] and emphasizes the environmental pathogenicity of *K. pneumoniae*. Consequently, farms should prioritize maintaining clean and disinfected livestock and poultry enclosures. Furthermore, the 62 *K. pneumoniae* isolates exhibited considerable genetic diversity, as evidenced by their categorization into 30 distinct *wzi* allele types and 35 different STs. This diversity underscores the complex nature of animal-derived *K. pneumoniae* strains in Xinjiang. We found a lack of correspondence between capsular serotypes and sequence types, as evidenced by strains with identical *wzi* alleles but differing STs (e.g., ST290 and ST1524 are wzi592), which aligns with previous findings [39]. Of particular concern is the identification of ST42 and wzi33-KL64 in both dairy- and swine-origin isolates, and wzi31-KL31-K31 in isolates from both dairy and chicken farms, suggesting potential cross-species transmission of *K. pneumoniae*. Such inter-species and regional transmission pose a significant risk and underscores the importance of implementing stringent infection control measures to prevent transmission and mitigate infection risks. 

The pathogenicity of *K. pneumoniae* relies heavily on its virulence factors, which are integral to its ability to cause disease [7]. In this study, virulence-related genes *entB, uge, wabG, fimH, mrkD*, and *ureA* were detected at high frequencies, and these virulence genes were also detected at high frequencies in *K. pneumoniae* isolates from Hubei Province, China [27]. Notably, the presence of the *allS* gene, involved in adrenergic metabolism regulation and crucial for *K. pneumoniae* virulence in liver infections, was only observed in isolates from cattle farms, albeit at a low detection rate. The *kfu* gene was identified in isolates from both dairy and chickens, suggesting a regional epidemic of *kfu* in Xinjiang. Although various virulence genes were detected in *K. pneumoniae* isolates from Xinjiang, genes associated with high virulence, such as *rmpA, magA, iucA*, and *iroN*, were not detected. It is hypothesized that the *K. pneumoniae* of animal origin now prevalent in Xinjiang mainly belongs to the cKP type. In contrast, *K. pneumoniae* carrying high virulence genes was detected in isolates from dairy sources in Hubei Province, Shandong Province, and swine and chicken sources in Henan Province, China, which implies that strains from Xinjiang exhibit relatively weak virulence compared with strains from other regions of China [27,40,41]. The virulence genes carried by *K. pneumoniae* were correlated with antibiotic resistance genes and antibiotic resistance phenotypes. In this study, we found that the iron carrier-associated gene *kfu* was correlated with multiple antibiotic resistance phenotypes, and it showed a low negative correlation with the resistance rate of AMC and a low positive correlation with the resistance rates of ENR and CIP. Among *K. pneumoniae* isolates from Jiangsu Province, China, a high positive rate of *kfuBC* was found to be negatively correlated with resistance to tetracycline, piperacillin, and streptomycin [42]. In summary, it can be hypothesized that *kfu* is important in regulating multi-drug resistance in *K. pneumoniae*. In addition, *fimH* was found to have a low positive correlation with the β-lactam resistance gene *bla*_SHV_, and *wabG*, *mrkD*, and *ureA* were found to have a low positive correlation with the quinolone resistance gene *oqxA* by comparison. Clonal hospital isolates of *K. pneumoniae* carrying carbapenemase genes of “high-risk” ST 15, 48, 101, 147, and 383 were found to contain hybrid plasmids with multiple antibiotic resistance and virulence genes [43]. Current studies suggest that plasmids play a key role in the transmission of genetic elements associated with antibiotic resistance and virulence. These plasmids have a variety of mobile genetic elements that are capable of frequent gene transcription, leading to plasmid fusions, which have led to increasing reports of carbapenem-resistant, highly virulent *K. pneumoniae* [44]. This finding is worrisome, indicating that virulence genes and resistance genes can interact with each other, and we will conduct subsequent studies on the interaction between virulence genes and resistance genes. 

In recent years, the prevalence of MDR *K. pneumoniae* of animal origin has been on the rise, posing a significant challenge to antimicrobial treatment [45]. Our study revealed varying levels of antibiotic resistance among *K. pneumoniae* isolated from different animal farms. Notably, all *K. pneumoniae* isolates demonstrated complete resistance to ampicillin, indicating the ineffectiveness of this antibiotic against these strains. Furthermore, high resistance to sulfisoxazole was observed across all isolates, likely attributable to the historical use of sulfonamide antibiotics as feed additives in livestock and poultry farming [46]. Carbapenems are not approved for use in food animals in China, which is consistent with the fact that all isolates in this experiment were highly susceptible to meropenem and imipenem. However, carbapenems are widely used in human medicine and clinical practice, and at the same time, their antibiotic resistant genes are capable of transferring and spreading in humans, animals, and the environment, and carbapenem-resistant *K. pneumoniae* is increasingly being reported in a variety of animals [47]. Resistance to amoxicillin/clavulanic acid was also notable, particularly among swine-origin isolates, contrasting with findings from a study in Thailand where *K. pneumoniae* isolates from slaughtered pigs were susceptible to this antibiotic [48]. Our study detected an MDR *K. pneumoniae* isolation rate of 48.39%, with swine farms exhibiting the highest percentage of MDR strains (66.66%), followed by chicken farms, dairy farms, and sheep farms. This phenomenon was also seen in *K. pneumoniae* isolates from Henan Province, China [41]. This highlights the seriousness of antibiotic resistance in Chinese swine farms, the cause of which may be related to irrational medication use on swine farms and incomplete disinfection of the farm environment and automated equipment; there is an urgent need for veterinarians and farmers to prioritize this issue and implement strict antibiotic use protocols and proper disinfection of farms.

Animals are recognized as reservoir hosts for Extended-spectrum β-lactamase (ESBL) and AmpC β-lactamase (AmpC), producing *K. pneumoniae* [49], with ESBL-associated resistance genes such as *bla*_SHV_, *bla*_TEM_, and *bla*_CTX-M_ often harbored within mobile genetic elements and capable of widespread transmission among humans, animals, and the environment [50]. Our study detected higher levels of *bla*_TEM_ and *bla*_SHV_ genes in different animal farms, which is consistent with the results of studies in other regions of China [27]. In China, *KPC*-type carbapenemases are more prevalent than other carbapenemases (e.g., *NDM* and *OXA-48* types) [51]. However, these common carbapenemase genes were not detected in our experiments, and only a small number of *bla*_VIM_ genes were detected in cattle, sheep, and pig farms. Correlation analysis was performed and revealed that there was no correlation between β-lactam resistance phenotypes and resistance genes, whereas Wu et al. [27] found a moderate positive correlation between EFT, CRO, and *bla*_CTX-M-15_, and between AMC and *bla*_OXA-1_ in isolates from Hubei, China. The reason for this discrepancy may be due to the different resistance genes and antibiotic drug classes tested in our study. Mechanisms of quinolone resistance include mutations in the quinolone resistance determining region (QRDR), acquisition of plasmid-mediated quinolone resistance (PMQR) genes, overexpression of efflux pumps, etc. [52]. In this study, the detection rate of the QRDR gene *gyrA* was 100%, while among the PMQR genes, *oqx AB* had the highest detection rate, and both *qnrA* and *aac(6′)-lb-cr* had less than 10.00% detection rate. This result is inconsistent with Kareem et al. [53] who found that the most common PMQR gene was *aac(6′)-Ib-cr* (92.50%). This may be a result of different molecular characterizations of prevalent *K*. *pneumoniae* due to geographical location. No significant association was found to exist between quinolone resistance phenotypes and resistance genes; this result is similar to that of Wu et al. [27]. It is hypothesized that there is a wide variety of quinolone resistance genes and there may be unknown genes, and methods such as whole-genome sequencing can be used to explore the correlation between them more clearly. Interestingly, we found a correlation between some β-lactam resistance phenotypes, quinolone resistance phenotypes, and antibiotic resistance genes of other classes. In the present study, AMC was found to have a low negative correlation (r = −0.306) with the quinolone resistance gene *oqxA*. Whereas, it has been found that the patient’s past use of quinolones or AMC promotes the development of AMC resistance and that plasmid-mediated quinolone resistance may drive the clonal spread of AMC resistance [54]. This is similar to our results, and it is hypothesized that it may be caused by the coexistence of multiclass antibiotic resistance genes on the plasmid and the co-mediated resistance of *K*. *pneumoniae* to multiple classes of antibiotics, and the exact mechanism of resistance needs to be further investigated. In animal husbandry, aminoglycosides are often used in combination with β-lactam antibiotics, and enzyme production is the main mechanism by which *K. pneumoniae* develops resistance [55]. We detected multiple aminoglycoside-modifying enzyme genes (*aadA1* and *aacC2*) in our isolates. Correlation analysis showed that both CN and KAN were positively correlated with *aacC2*, and it was hypothesized that the *aacC2* gene might be involved in the resistance of *K*. *pneumoniae* to aminoglycosides. Among the 62 *K*. *pneumoniae* strains, the sulfonamide resistance genes *sul1* and *sul2* were detected at a high rate, and correlation analysis showed that SF and SXT were positively correlated with *sul2*, and the resistance of *K*. *pneumoniae* to sulfisoxazole and cotrimoxazole might be mediated by *sul2*. The tetracycline resistance gene *tetA* had a higher detection rate (41.94%), and TCY was positively correlated with both *tetA* and *tetB*, and *K*. *pneumoniae* resistance to tetracycline might be mediated by *tetA* and *tetB*. In addition, the detection rate of *floR* was also high, which was consistent with the study of Nobrega et al. [56]. Meanwhile, the analysis revealed a moderate positive correlation between FFC and *floR*, and the resistance of *K*. *pneumoniae* to florfenicol may be mediated by *floR*. Although the chloramphenicol derivative florfenicol is only used for animal infections, plasmids carrying the *floR* gene can be transmitted among Enterobacteriaceae bacteria, thus posing a potential risk of human–animal cross-infection [57].

## 5. Conclusions

The threat of *K. pneumoniae* to poultry and livestock cannot be ignored. This study revealed that *K. pneumoniae* strains from different animal sources in Xinjiang differed in virulence gene and resistance gene carriage, and revealed a worrying phenomenon of multi-drug resistance, which was particularly severe in pig and chicken farms. We also found a potential link between virulence and resistance genes and antibiotic resistance phenotypes. In addition, by typing *K. pneumoniae*, we found that there may be a risk of cross-species and cross-regional transmission between different animal sources. Therefore, the epidemiologic investigation and surveillance of *K. pneumoniae* in different animal sources should be further expanded, and the interaction between virulence genes and antibiotic resistance should be more thoroughly investigated.

## Figures and Tables

**Table 1:** Sample collection information.

Farm Types	Total Number of Samples	Types of Samples	Number of Samples
Dairy farm A	88	milk from cows with CM	10
		milk from cows with SCM	13
		nasal swab of healthy cows	31
		anal swab of healthy cows	5
		anal swab of calves	10
		nasal swab of calves	10
		feed	3
		bedding	6
Dairy farm B	41	nasal swab of healthy cows	13
		anal swab of healthy cows	10
		anal swab of calves	8
		nasal swab of calves	8
		feed	1
		bedding	1
Chicken farm	76	anal swab of laying hens	69
		environmental samples	7
Sheep farm	40	nasal swabs of sheep with respiratory disease	34
		environmental samples	6
Swine farm	66	nasal swabs of pig with respiratory disease	53
		environmental samples	13

**Table 2:** Percentage of virulence genes of *K. pneumoniae* from different animal farms.

Types	Virulence Gene	Positive Rates (%)	No. of Positive Samples/Total (%) from Farm			
			Dairy	Chicken	Sheep	Pig
regulation	*rmpA*	0.00	0/43(0.00)	0/11(0.00)	0/5(0.00)	0/3(0.00)
	*magA*	0.00	0/43(0.00)	0/11(0.00)	0/5(0.00)	0/3(0.00)
lipopolysaccharides	*uge*	88.71	37/43(86.07)	10/11(90.91)	5/5(100.00)	3/3(100.00)
	*wabG*	98.39	43/43(100.00)	11/11(100.00)	4/5(80.00)	3/3(100.00)
iron uptake	*entB*	98.39	42/43(97.67)	11/11(100.00)	5/5(100.00)	3/3(100.00)
	*kfu*	20.97	8/43(18.60)	5/11(45.45)	0/5(0.00)	0/3(0.00)
	*iroN*	0.00	0/43(0.00)	0/11(0.00)	0/5(0.00)	0/3(0.00)
	*icuA*	0.00	0/43(0.00)	0/11(0.00)	0/5(0.00)	0/3(0.00)
fimbriae	*fimH*	90.32	40/43(93.02)	10/11(90.91)	4/5(80.00)	2/3(66.67)
	*mrkD*	95.16	42/43(97.67)	10/11(90.91)	4/5(80.00)	3/3(100.00)
urease	*ureA*	98.39	43/43(100.00)	11/11(100.00)	4/5(80.00)	3/3(100.00)
allantoin	*allS*	1.61	1/43(2.33)	0/11(0.00)	0/5(0.00)	0/3(0.00)

Note: Positive rates = (total number of genes detected/total number of *K. pneumoniae* isolates) × 100%.

**Table 3:** Percentage resistance rate of *K. pneumoniae* in different animal farms.

Antibiotics	No. of Resistant Samples/Total (%) from Farm			
	Dairy	Chicken	Sheep	Pig
Florfenicol	8/43(18.60)	8/11(72.73)	1/5(20.00)	2/3(66.67)
Enrofloxacin	24/43(55.81)	7/11(63.64)	0/5(0.00)	1/3(33.33)
Ciprofloxacin	5/43(11.63)	6/11(54.55)	0/5(0.00)	0/3(0.00)
Ceftazidime	2/43(4.65)	0/11(0.00)	1/5(20.00)	0/3(0.00)
Ceftriaxone	7/43(16.28)	5/11(45.45)	1/5(20.00)	1/3(33.33)
Cefotaxime	6/43(13.95)	4/11(36.36)	1/5(20.00)	1/3(33.33)
Ampicillin	43/43(100.00)	11/11(100.00)	5/5(100.00)	3/3(100.00)
Amoxicillin/clavulanic acid	26/43(60.47)	5/11(45.45)	5/5(100.00)	2/3(66.67)
Sulfisoxazole	24/43(55.81)	10/11(90.91)	1/5(20.00)	3/3(100.00)
Sulfamethoxazole/trimethoprim	7/43(16.28)	8/11(72.73)	1/5(20.00)	2/3(66.67)
Gentamicin	8/43(18.60)	8/11(72.73)	2/5(40.00)	2/3(66.67)
kanamycin	4/43(9.30)	6/11(54.55)	2/5(40.00)	2/3(66.67)
Tetracycline	7/43(16.28)	8/11(72.73)	1/5(20.00)	2/3(66.67)
Imipenem	0/43(0.00)	0/11(0.00)	0/5(0.00)	1/3(33.33)
Meropenem	0/43(0.00)	0/11(0.00)	0/5(0.00)	0/3(0.00)

**Table 4:** Percentage of antibiotic resistance genes of *K. pneumoniae* from different animal farms.

Types	Resistance Gene	Positive Rates (%)	No. of Positive Samples/Total (%) from Farm			
			Dairy	Chicken	Sheep	Pig
β-Lactams	*bla*_IMP_	0.00	0/43(0.00)	0/11(0.00)	0/5(0.00)	0/3(0.00)
	*bla*_VIM_	11.29	4/43(9.30)	0/11(0.00)	2/5(40.00)	1/3(33.33)
	*bla*_OXA-48_	0.00	0/43(0.00)	0/11(0.00)	0/5(0.00)	0/3(0.00)
	*bla*_NDM_	0.00	0/43(0.00)	0/11(0.00)	0/5(0.00)	0/3(0.00)
	*bla*_KPC_	0.00	0/43(0.00)	0/11(0.00)	0/5(0.00)	0/3(0.00)
	*bla*_DHA_	0.00	0/43(0.00)	0/11(0.00)	0/5(0.00)	0/3(0.00)
	*bla*_FOX_	0.00	0/43(0.00)	0/11(0.00)	0/5(0.00)	0/3(0.00)
	*bla*_CTX-M-2_	8.06	2/43(4.65)	1/11(9.09)	1/5(20.00)	1/3(33.33)
	*bla*_SHV_	75.81	34/43(79.07)	7/11(63.63)	3/5(60.00)	3/3(100.00)
	*bla*_TEM_	79.03	39/43(90.70)	5/11(45.45)	3/5(60.00)	2/3(66.67)
Quinolones	*oqxA*	87.10	42/43(97.67)	8/11(72.73)	2/5(40.00)	2/3(66.67)
	*aac(6′)-Ib-cr*	4.84	1/43(2.33)	1/11(9.09)	1/5(20.00)	0/3(0.00)
	*qnrA*	9.68	1/43(2.33)	2/11(18.18)	2/5(40.00)	1/3(33.33)
	*gyrA*	100.00	43/43(100.00)	11/11(100.00)	5/5(100.00)	3/3(100.00)
Amphenicols	*floR*	48.39	18/43(41.86)	7/11(63.63)	3/5(60.00)	2/3(66.67)
Sulfonamides	*sul1*	50.00	21/43(48.84)	6/11(54.55)	2/5(40.00)	2/3(66.67)
	*sul2*	41.94	14/43(32.56)	7/11(63.63)	3/5(60.00)	2/3(66.67)
Tetracyclines	*tetA*	41.94	15/43(34.88)	7/11(63.63)	2/5(40.00)	2/3(66.67)
	*tetB*	8.06	1/43(2.33)	2/11(18.18)	1/5(20.00)	1/3(33.33)
Aminoglycosides	*aadA1*	38.71	19/43(44.19)	4/11(36.37)	1/5(20.00)	0/3(0.00)
	*aacC2*	19.35	6/43(13.95)	2/11(18.18)	2/5(40.00)	2/3(66.67)

Note: Positive rates = (total number of genes detected/total number of *K. pneumoniae* isolates) × 100%.

## Data Availability

The datasets used and/or analyzed during the current study are available from the corresponding author upon reasonable request.

## Supplementary Materials

The following supporting information can be downloaded at: https://www.mdpi.com/article/10.3390/ani14101433/s1. Table S1: Primer sequences of *wzi* capsular serotyping and MLST; Table S2: Primer sequences of virulence genes and resistance genes; Table S3: Analysis of *wzi* Allele Types for 62 *K. pneumoniae* isolates; Table S4: Analysis of MLST for 62 *K. pneumoniae* isolates. Figure S1: Electrophoresis of PCR products of *khe* gene of 62 isolates; Figure S2: Blast of *K. pneumonia* in the NCBI database.
