# Supplementary material for: Epidemiological, Virulence, and Antibiotic Resistance Analysis of Klebsiella pneumoniae, a Major Source of Threat to Livestock and Poultry in Some Regions of Xinjiang, China
Source: Animals (Basel). 2024 May 10;14(10):1433. doi: 10.3390/ani14101433 (PMC11117231; doi:10.3390/ani14101433)
Supplement: Supplementary file 1 [file animals-14-01433-s001.zip › animals-2984583-supplementary.pdf]

Table S1: Primer sequences of *wzi* capsular serotyping and MLST.

Gene	Sequence（5′-3′）	Products /bp	annealing temperature /℃
wzi	F：GTGCCGCGAGCGCTTTCTATCTTGGTATTCC<br>R：GAGAGCCACTGGTTCCAGAACTTGACCGC	580	60
rpoB	F：GGCGAAATGGCWGAGAACCA<br>R：GAGTCTTCGAAGTTGTAACC	1075	50
gapA	F：TGAAATATGACTCCACTCACGG<br>R：CTTCAGAAGCGGCTTTGATGGCTT	662	60
mdh	F：CCCAACTCGCTTCAGGTTCAG<br>R：CCGTTTTTCCCCAGCAGCAG	756	50
pgi	F：GAGAAAAACCTGCCTGTACTGCTGGC<br>R：CGCGCCACGCTTTATAGCGGTTAAT	566	50
phoE	F：ACCTACCGCAACACCGACTTCTTCGG<br>R：TGATCAGAACTGGTAGGTGAT	602	50
infB	F：CTCGCTGCTGGACTATATTCG<br>R：CGCTTTCAGCTCAAGAACTTC	462	50
tonB	F：CTTTATACCTCGGTACATCAGGTT<br>R：ATTCGCCGGCTGRGCRGAGAG	539	45

Table S2: Primer sequences of virulence genes and resistance genes.

Gene	Sequence（5′-3′）	Products /bp	annealing temperature/℃
fimH	F：GTCTACGTTAACCTGACCCCG<br>R：ATTGATAGACAAAGGTGATGCCGAT	781	56
mrkD	F：AAGCTATCGCTGTACTTCCGGCA<br>R：GGCGTTGGCGCTCAGATAGG	340	60
uge	F：TCTTCACGCCTTCCTTCACT<br>R：GATCATCCGGTCTCCCTGT	534	53
wabG	F：CTCTGGTGCGGCAGAAGTAC<br>R：TGGCCGTCGACGATAAACTC	931	52
rmpA	F：ACCCTTTACAGCCAAATTTTCTTGT<br>R：CTGGGCTACCTCTGCTTCATAT	468	52
magA	F：TGATAAGTGGCGGAGATTCTGA<br>R：TGATAAGTGGCGGAGATTCTGA	542	52
iroN	F：GAATGAAACTACCGCCCCCA<br>R：TGTGGAGTGGAGGCGAGATA	1033	54
entB	F：ATTTCCTCAACTTCTGGGGC<br>R：AGCATCGGTGGCGGTGGTCA	371	60
iucA	F：GCTTATTTCTCCCCAACCC<br>R：TCAGCCCTTTAGCGACAAG	583	59
kfu	F：GAAGTGACGCTGTTTCTGGC<br>R：TTTCGTGTGGCCAGTGACTC	797	55
allS	F：CTTCAGCAGATAAATGACGGGGTAG<br>R：GTGGGTAAACCGCCATATTTTCC	244	56
ureA	F：GCTGACTTAAGAGAACGTTATG<br>R：GATCATGGCGCTACCT(C/T)A	337	55

blaCTX-M-2	F：AAATGTGCTGCTCCTTTCGTGAGC<br>R：AGGGTTCGTTGCAAGACAAGACTG	1122	55
blaSHV	F：ATGCGTTATATTCGCCTGTG<br>R：TGCTTTGTTATTCGGGCCAA	747	55
blaTEM	F：TCGCCGCATACACTATTCTCAGAATGA<br>R：ACGCTCACCGGCTCCAGATTTAT	445	55
blaIMP	F：GGAATAGAGTGGCTTAAYTCTC<br>R：GGTTTAAYAAAACAACCACC	232	55
blaVIM	F：GATGGTGTTTGGTCGCATA<br>R：CGAATGCGCAGCACCAG	390	55
blaOXA-48	F：GCGTGGTTAAGGATGAACAC<br>R：CATCAAGTTCAACCCAACCG	438	55
blaNDM	F：GGTTTGGCGATCTGGTTTTC<br>R：CGGAATGGCTCATCACGATC	621	55
blaKPC	F：CGTCTAGTTCTGCTGTCTTG<br>R：CTTGTCATCCTTGTTAGGCG	798	55
blaDHA	F：GCCTGTTTGGTGCTCTGA<br>R：GCACGGTTATACGGCTGA	460	55
blaFOX	F：AACATGGGGTATCAGGGAGATG<br>R：CAAAGCGCGTAACCGGATTGG	190	55
oqxA	F：CTCGGCGCGATGATGCT<br>R：CCACTCTTCACGGGAGACGA	392	55
aac(6')-Ib-cr	F：TTGCGATGCTCTATGAGTGGCTA<br>R：CTCGAATGCCTGGCGTGTTT	482	55
qnrA	F：TCAGCAAGCGGATTTCTCA<br>R：GGCAGCACTATTACTCCCA	608	55
gyrA	F：CGCGTACTATACGCCATGAACGTA<br>R：ACCGTTGATCACTTCGGTCAGG	420	55
aadA1	F：TATCCAGCTAAGCGCGAAC<br>R：ATTTGCCGACTACCTTGGT	447	55
aacC2	F：ATGCATACGCGGAAGGCAATAAC<br>R：CTAACCGGAAGGCTCGCAAG	861	55
tetA	F：GTGAAACCCAACATACCCC<br>R：GAAGGCAAGCAGGATGTAG	888	53
tetB	F：CCTCAGCTTCTCAACGCGTG<br>R：GCACCTTGCTGATGACTCTT	634	55
sul1	F：GTGACGGTGTTCGGCATTCT<br>R：TCCGAGAAGGTGATTGCGCT	779	55
sul2	F：CGGCATCGTCAACATAACCT<br>R：TGTGCGGATGAAGTCAGCTC	721	55
floR	F：TTTGGTCCGCTCTCAGAC<br>R：CGAGAAGAAG(A\G)CGAAGAAG	496	53

Table S3. Analysis of *wzi* Allele Types for 62 *K. pneumoniae* isolates.

**wzi allele types**	**Proportion (%)**	**No. of isolates from farm**			
		**Dairy**	**Chicken**	**Sheep**	**Pig**
wzi46-KL61KL13-K46K61	8.06	5	0	0	0
wzi42-KL42-K42	6.45	4	0	0	0
wzi31-KL31-K31	6.45	2	2	0	0

wzi21-KL21-K21	4.84	3	0	0	0
wzi50-KL15KL17KL51KL52-K15K17K50K51K52	4.84	3	0	0	0
wzi130-KL58	4.84	3	0	0	0
wzi355-KL114	4.84	0	3	0	0
wzi33-KL64	4.84	2	0	0	1
wzi114-KL30KL2KL8KL111-K30	3.23	2	0	0	0
wzi385-KL22KL37	3.23	2	0	0	0
wzi592	3.23	2	0	0	0
wzi11-KL11-K11	1.61	1	0	0	0
wzi82-KL23KL18KL38-K23	1.61	1	0	0	0
wzi102-K31	1.61	1	0	0	0
wzi135-KL3-K3	1.61	1	0	0	0
wzi162-KL122-K35	1.61	1	0	0	0
wzi202-KL127KL155	1.61	1	0	0	0
wzi216-KL119	1.61	1	0	0	0
wzi274-KL30	1.61	1	0	0	0
wzi354-KL114	1.61	1	0	0	0
wzi447-KL124	1.61	1	0	0	0
wzi464	1.61	1	0	0	0
wzi472	1.61	1	0	0	0
wzi605	1.61	1	0	0	0
wzi645	1.61	1	0	0	0
wzi17-KL7-K7	1.61	0	0	1	0
wzi147-K79	1.61	0	0	1	0
wzi112-KL13KL39-K19	1.61	0	0	1	0
wzi43-KL43-K43	1.61	0	1	0	0
wzi19-KL19-K19	1.61	0	0	0	1
unknowns	14.52	1	5	2	1

Table S4. Analysis of MLST for 62 *K. pneumoniae* isolates.

ST	Proportion (%)	No. of isolates from farm			
		Dairy	Chicken	Sheep	Pig
791	6.45	4	0	0	0
5387	6.45	0	4	0	0
42	4.84	2	0	0	1
49	3.23	2	0	0	0
161	3.23	2	0	0	0
5121	3.23	2	0	0	0
5179	3.23	2	0	0	0
5490	3.23	2	0	0	0
6085	3.23	0	2	0	0
17	1.61	1	0	0	0
60	1.61	1	0	0	0
76	1.61	1	0	0	0
200	1.61	1	0	0	0
290	1.61	1	0	0	0

322	1.61	1	0	0	0
774	1.61	1	0	0	0
776	1.61	1	0	0	0
1066	1.61	1	0	0	0
1337	1.61	1	0	0	0
1524	1.61	1	0	0	0
1540	1.61	1	0	0	0
1644	1.61	1	0	0	0
1832	1.61	1	0	0	0
2108	1.61	1	0	0	0
3030	1.61	1	0	0	0
3071	1.61	1	0	0	0
3839	1.61	1	0	0	0
4426	1.61	1	0	0	0
4568	1.61	1	0	0	0
5056	1.61	1	0	0	0
5154	1.61	1	0	0	0
967	1.61	0	0	0	1
2455	1.61	0	1	0	0
347	1.61	0	0	1	0
4844	1.61	0	0	1	0
unknowns	20.97	5	4	3	1

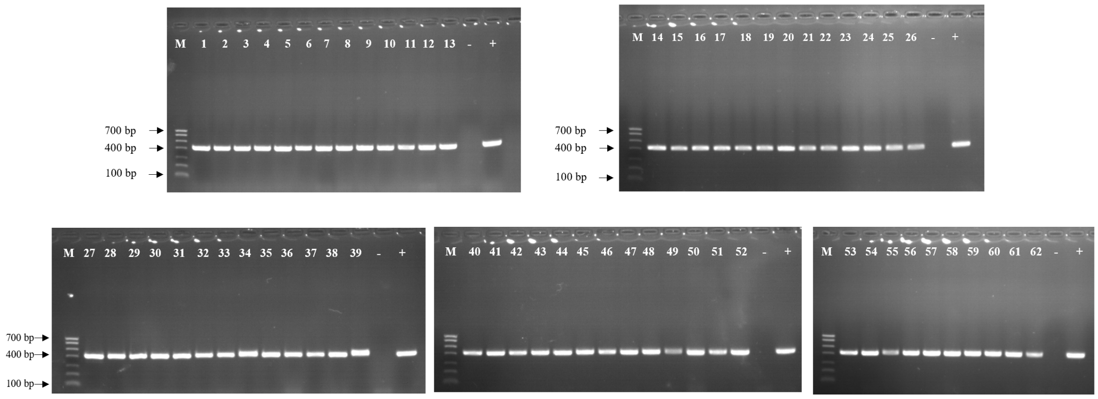

Figure S1: Electrophoresis of PCR products of *khe* gene of 62 isolates. M = molecular weight marker; – = negative control (water); + = positive control (*K. pneumoniae* CMCC 46117); 1-62 = PCR product of the *khe* gene of isolates.

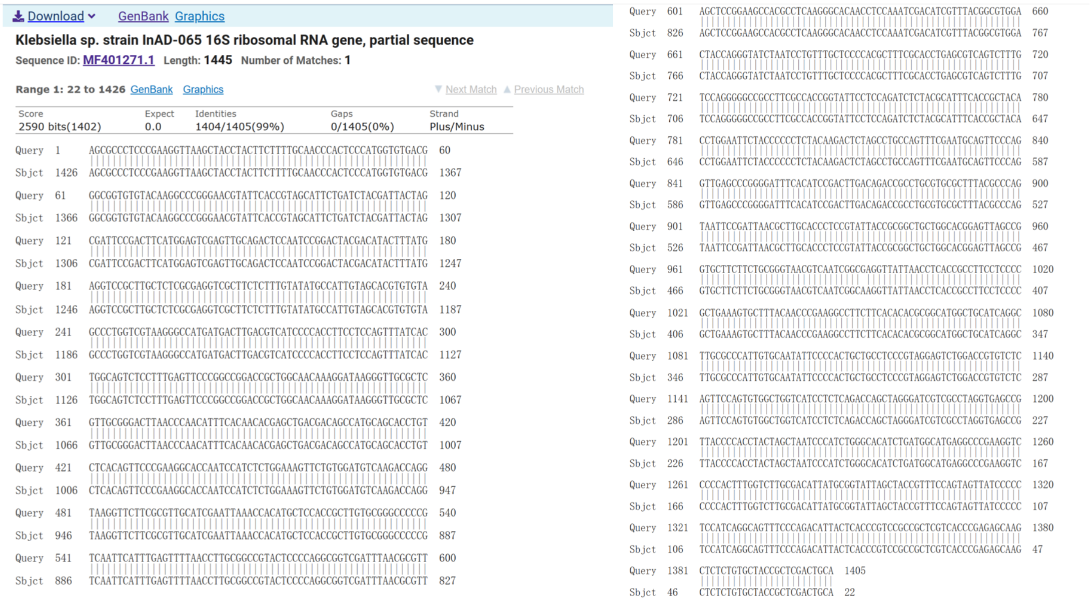

Figure S2. Blast of *K. pneumonia* in the NCBI database
